# The Optimal Axial Anatomical Site for a Single-Slice Area to Quantify the Total Volume of Visceral Adipose Tissue in Quantitative CT

**DOI:** 10.3389/fendo.2022.870552

**Published:** 2022-06-23

**Authors:** Sihui Chen, Duoshan Ma, Danyang Su, Yali Li, Xi Yu, Yaojun Jiang, Jianbo Gao, Yan Wu

**Affiliations:** Department of Radiology, The First Affiliated Hospital of Zhengzhou University, Zhengzhou, China

**Keywords:** optimal anatomical axial site, volume prediction equation, visceral adipose tissue, QCT, total VAT

## Abstract

**Purpose:**

Determine the association between cross-sectional visceral adipose tissue (VAT) area of different anatomic locations and total abdominopelvic VAT volume; identify the optimal measurement site in a single-slice to quantify the total VAT volume.

**Method:**

Participants who underwent non-contrast abdominal scan by quantitative CT (QCT) were enrolled from May 2021 to October 2021. The VAT area (cm^2^) at different anatomic sites as upper-pole, lower-pole, and hilum of the kidney, intervertebral disc of L2/L3 and L5/S1, and umbilical level were measured on QCT PRO BMD workstation (Mindways QCT PRO workstation). The total VAT volume (cm^3^) from the upper pole of kidney to the L5/S1 intervertebral disc of the pelvis (abdominopelvic region) was obtained by using Siemens Healthineers Syngo *via* Frontier cardiac risk assessment. Regression models were used to identify the optimal single-slice in different gender for estimating VAT volume. Statistical significance was established at *P* < 0.05.

**Results:**

Total of 311 Chinese participants including 179 men [age, 55.1 ± 14.9 years; body mass index (BMI), 24.2 ± 3.2 kg/m^2^; total VAT volume, 2482.6 ± 1276.5 mL] and 132 women [age, 54.3 ± 14.9; BMI, 23.5 ± 2.9; total VAT volume, 1761.5 ± 876.4]. Pearson’s correlation analysis revealed a strong association between the VAT area and total abdominopelvic VAT volume at the hilum of the kidney in both men (*r=*0.938, *P*<0.001) and women (*r*=0.916, *P*<0.001). Adjust for covariates including age, BMI, and waist circumference make a relatively small effect on predicting the total VAT volume.

**Conclusions:**

Measurement of cross-sectional areas at the hilum of the kidney in both genders showed a strongest relation to TVAT volume. Our results may provide an identifiable and valuable axial landmark for measuring visceral adipose tissue in clinical practice.

## Introduction

The excess accumulation of abdomen fat has become a global health issue and has increased the economic burden on society (1–4). Obesity is frequently defined by indirectly anthropometrical indicators like body mass index (BMI), waist circumference (WC), and waist-to-hip ratio (WHR). Although BMI provides an obviously useful population-level measure for overweight and obesity than WC and WHR, it should be considered as a rough guide because BMI may not correspond to the same degree of fatness in different individuals. Meanwhile, anthropometrics can’t reflect the fat distribution and differentiate visceral adipose tissue (VAT) and subcutaneous adipose tissue (SAT) (5, 6). The measurement of single-slice visceral adipose allows better characterization of body fat distribution than BMI, especially the VAT in the abdomen. Moreover, previous studies indicated that visceral fat is highly associated with cardiometabolic risk, insulin resistance, type 2 diabetes mellitus (2, 7–9). The measurement site for VAT has a substantial influence on the assessment of both cardiac and metabolic syndrome risk (10).

Abdominal fat quantifications can be measured accurately using computed tomography (CT) (11–14), quantitative CT (QCT) (15–17), magnetic resonance imaging (MRI) (18, 19), and bio-impedance analysis (BIA) (17). However, CT scan delivers radiation dosage exposure on patients and MRI in the assessment of VAT quantification is restricted by its time consuming and high costs. QCT may have relatively better performance than BIA in identifying individuals at high risk of visceral obesity-related health conditions (17). Thus, establishing a practical alternative method to estimate abdominal fat content is urgently needed. The usefulness of QCT software for volumetric bone mineral density (BMD) analysis has been widely recognized, and the supplement tool of abdominal fat quantitative analysis achieved favorable results in opportunistic scanning uses without additional radiation exposure (16, 20). It is feasible to investigate the abdominal VAT in a QCT scan.

The VAT instead of SAT volume has a better indicative effect on metabolic syndrome (21, 22) which independently associated with some inflammatory markers (23). However, the evaluation of total VAT volume (TVAT) was approximately equal to the area of VAT times thickness in preceding studies (11, 15). Research indicates that there might be misinterpretation in the process of calculating the TVAT volume (11). Most investigators use L4/L5 as the common location for measuring VAT (11, 24), since a single axial CT image of VAT be able to reflect TVAT volume and narrow the scanning range of CT and reduce the examination time and costs on MRI. While other studies (10, 15, 18) reported that the selected image in upper abdomen has a greater deposition of the more metabolically-active VAT. Furthermore, Kuk et al. (10) concluded that VAT area obtained at L1/L2 or L2/L3 may be a better indicator of metabolic syndrome than measurements obtained at L4/L5. To sum up, these observations suggest that there is a stronger correlation between VAT area of the upper abdomen and metabolic syndrome or cardiometabolic disease, which is suitable for total VAT volume quantitative estimation. Until now, few study has utilized QCT-derived VAT area measurement based on identifiable and easy-to-detecting anatomical landmarks in Chinese population. Therefore, this study aimed to investigate the optimal anatomical landmark for measuring VAT area in QCT images to quantify total VAT volume.

## Subjects and Methods

### Study Participants

Participants consisted of 325 asymptomatic Chinese individuals who had received non-contrast abdominal QCT scan performed on Philips Healthcare scanner (Brilliance iCT Elite FHD, OH, USA) and Siemens Healthineers scanner (Somatom Force, Erlangen, Germany) from May 2021 to October 2021. Participants with the following conditions were excluded: (I) with current systemic corticosteroids treatment; (II) known hyperthyroidism or hypothyroidism; (III) known abdominal surgery history; and (IV) BMI lower than 18.5 kg/m^2^. The study was approved by the ethics committees of institutional review board of the First Affiliated Hospital of Zhengzhou University and strictly adherence to HIPAA Privacy rule. All participants have written informed consent.

### Anthropometric Characteristics

The anthropometric characteristics of participants including age, sex, and BMI were collected. BMI is defined as a participant’s weight in kilograms divided by the square of the height in meters in adults (kg/m^2^) (25). Following the WHO BMI norms, the participants were divided into two groups of normal BMI (18.5<BMI<24.9 kg/m^2^) and excess BMI (overweight/obesity, BMI≥ 25 kg/m^2^).

### CT Scanning Protocol

The monthly quality control analysis of CT scanners was performed by qualified technologists before data acquisitions and the whole study duration using European Spine Phantom (ESP No.145) (Mindways Software, Austin, TX, USA). Detailed scan parameters were showed in **Table 1**. CT images were then transferred to QCT PRO workstation and Siemens Healthineers Syngo post-processing workstation, respectively.

**Table 1:** Summary of acquisition parameters of 2 CT scanners.

CT parameters	Brilliance iCT Elite	Somatom Force
Tube voltage (kV)	120	120
Tube current-time product (mAs)	automatic current selection (DoseRight Index-23)	anatomic tube current modulation (CARE Dose 4D)
Pitch (approximate number)	0.914	0.8
Detector configuration (mm)	128×0.625	96×0.6
Matrix size	512×512	512×512
Slice thickness/increment (mm)	1.0/1.0	1.0/1.0
Reconstruction kernel	Standard	Br40
DFOV (mm)	350	350
Acquisition mode	Helical	Helical
Gantry rotation times (s)	0.5	0.5
145.5	145.5

CT, computed tomography; DFOV, display field of view.

### The Measurement of Single-Slice VAT Area

**Figure 1:**
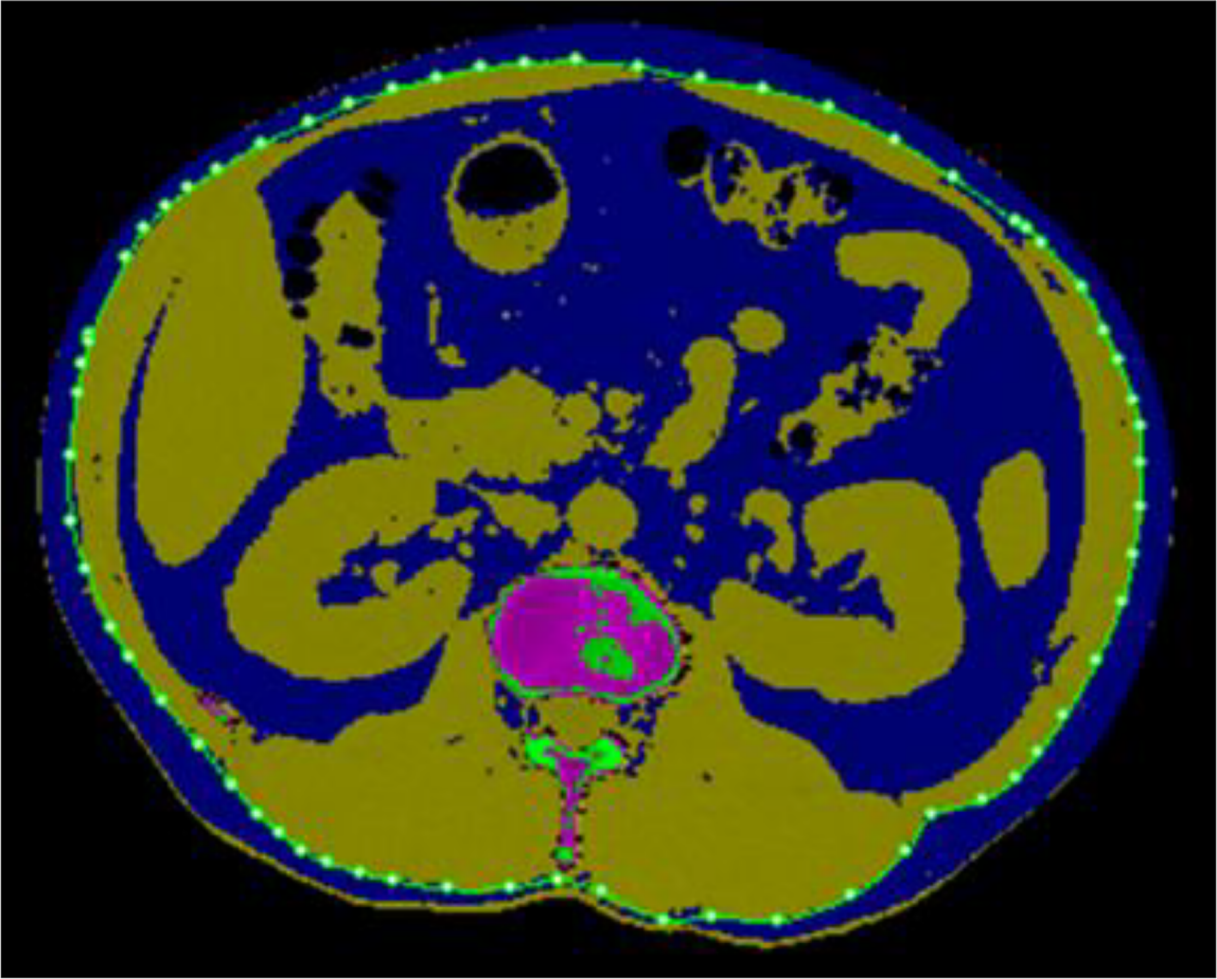
Anatomical location at the hilum of kidney where the maximum correlation of area to volume VAT is achieved for quantitative CT (QCT) method. An attenuation-based semi-automated segmentation using Tissue Composition and the fat was mapped as blue color, then the outer contour of the abdominal wall was used to differentiate visceral adiposity (inner) and subcutaneous visceral adiposity (outer).

Cheng et al. (15) researched the optimal anatomic site for a single slice to estimate the total volume of VAT at each intervertebral disc (T12/L1 to L5/S1) and the umbilical level. It suggested that a single slice at L2/L3 level performed best in the measurement of VAT area. The current study selected a more identifiable abdominal anatomical landmarks, such as the upper-, lower-pole, and hilum of the kidney, the intervertebral space of L2/L3 and L5/S1, and umbilical level based on their research. Image analysis was carried out using Mindways QCT PRO software version 6.1 (Mindways QCT PRO workstation). The VAT area (cm^2^) was measured using the “Tissue Composition Module-Beta 1.0” of the software, the CT value of -150 to -50 HU was semi-automatically segmented and represented in blue color, and then the outer contour of the abdominal wall was automatically outlined to separating between VAT and SAT (**Figure 1**). All the measurements were carried out by two trained and qualified radiologists.

### The Measurement of Total VAT Volume

**Figure 2:**
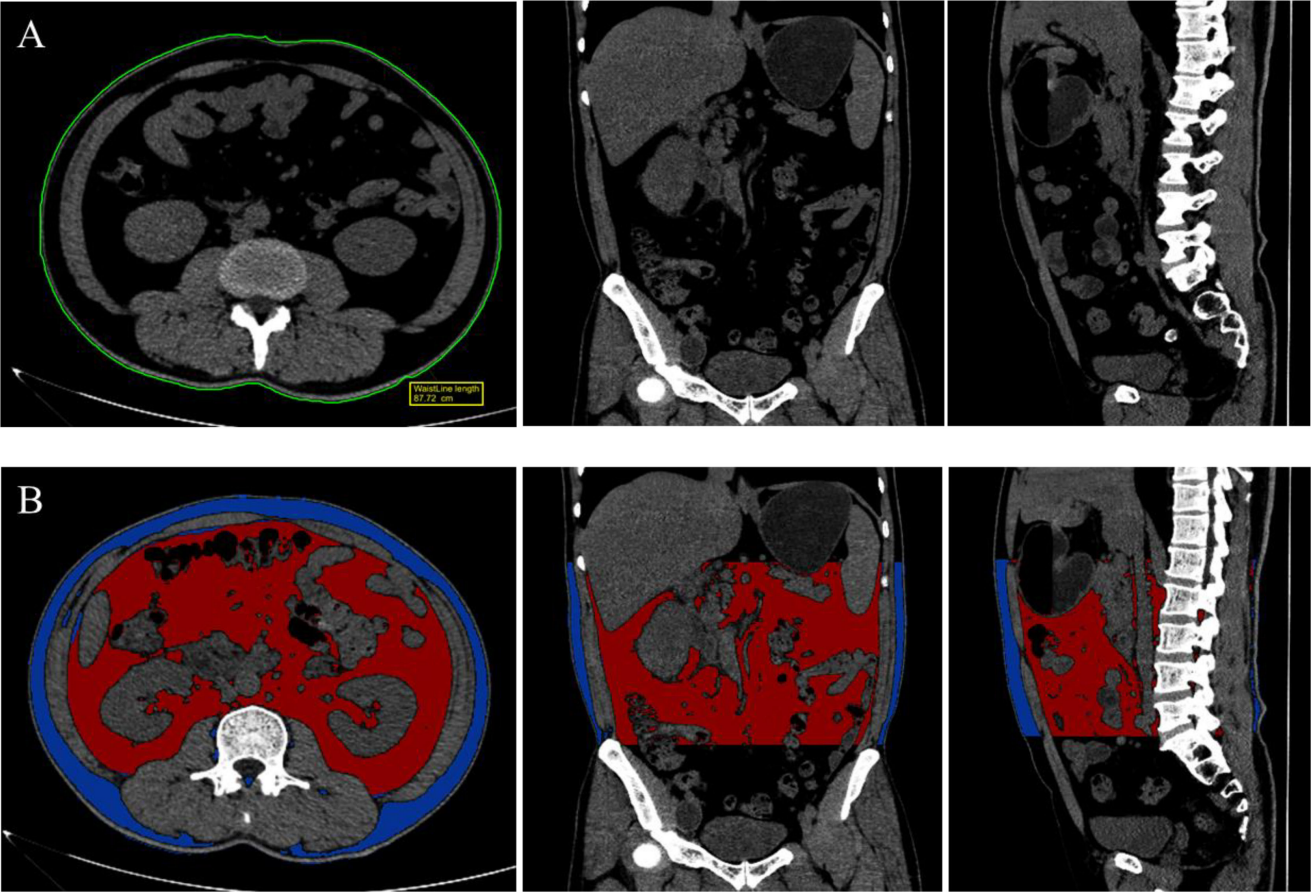
Measurement of waist circumference **(A)** at the middle of the lower rib margin and the iliac crest and Segmentation of visceral adiposity tissue **(B)**, ranging from the upper pole of kidney and L5/S1 intervertebral disc, both achieved in Siemens Healthineers Syngo *via* Frontier application with multi-planar reformation (MPR) images.

To obtain the overall abdominopelvic VAT volume, we utilized attenuation-based and semi-automatically segmentation methods with a cardiac risk assessment tool (Siemens Healthineers Syngo *via* Frontier, USA). The region of interest (ROI) of TVAT volume (cm^3^) was manually drawn along the abdominal inner wall to differentiate SAT and VAT with default thresholds (-150 to -50 HU). On the longitudinal axis, the abdominopelvic region ranges from the upper pole of kidney to the L5/S1 intervertebral disc (**Figure 2**). Finally, data were recorded as abdominopelvic TVAT volume (mL). WC was measured between the lower rib margin and the iliac crest in multi-planar reformation (MPR) images with all participants in a supine position. These data acquisitions were all achieved within Siemens Healthineers Syngo software application.

### Statistical Analysis

All statistical processing and analysis were performed on SPSS 21.0 (IBM Corp., Armonk, NY, USA) and GraphPad prism 8.0.2 (GraphPad Software, La Jolla, CA, USA). Continuous variables are represented as means (SD) or medians (interquartile range) depending on how the distribution was tested with the Shapiro-Wilks test. All variables were compared using two-sided Student’s *t*-test for gender and BMI differences. Pearson correlation coefficient (*r*) was performed to analyze the association between the single-slice VAT area and TVAT volume at each anatomical landmarks in terms of identifying the optimal anatomic site in total abdominopelvic VAT volume. The optimal anatomical sites for quantifying TVAT volume were then chosen as the best slice in linear regression models and model fitted predictions for TVAT volume estimation in men and women, respectively. Additionally, multiple linear regression model were used for the VAT area of the selected slice predicting the total VAT volume and adjusted for age, sex, waist circumference, and BMI. Data were considered significant when *P* < 0.05.

## Results

### Study Participants’ Basic Characteristics

A total of 311 participants (179 men, 55.3 ± 14.9 years, range, 18-93 years; 132 women, 54.3 ± 14.9 years, range, 19-87 years) were recruited in the study (**Table 2**). The mean BMI was 24.2 ± 3.2 kg/m^2^ in men, and 23.5 ± 2.9 kg/m2 in women. There were 201 participants in normal BMI (18.5-24.9kg/m^2^), whilst 110 participants in excess BMI (>25.0kg/m^2^). There were significant statistical differences in height, weight, BMI, WC, VAT area at different anatomic sites, and TVAT volume between men and women (*P<*0.05).

**Table 2:** Characteristics of the study population.

Variables (N, %)	Total (311)	Women (132, 42.4%)	Men (179, 57.6%)	*P* value*
Age (years)	54.9 ± 14.9	54.3 ± 14.9	55.3 ± 14.9	0.559
Height (m)	1.67 ± 0.07	1.61 ± 0.05	1.71 ± 0.05	<0.001*
Weight (kg)	66.8 ± 11.7	60.7 ± 9.1	71.4 ± 11.3	<0.001*
BMI (kg/m^2^)	23.9 ± 3.1	23.5 ± 2.9	24.2 ± 3.2	0.036*
WC (cm)	87.9 ± 9.8	85.3 ± 9.0	89.8 ± 9.9	<0.001*

P* for difference between gender groups; BMI, body mass index; WC, waist circumference.

### Comparison of Abdominal VAT Area and Total VAT

Overall, the VAT area at each anatomical location indicated a relatively higher abdominal visceral fat accumulation in men than in women (**Table 3**). The highest VAT area in men among these locations was located at the hilum of kidney (195.3 ± 83.0cm^2^), whilst in women was at the lower pole of kidney (140.8 ± 63.1cm^2^). **Table 4** showed QCT-derived VAT area and CT-derived TVAT volume results for normal and excess BMI subgroups between genders. Stratified by BMI, most normal BMI and excess BMI subgroups have a relatively higher VAT area at the hilum of kidney. But in excess BMI subgroup in women (BMI>25kg/m^2^), the highest VAT area was located at the lower pole of kidney. Significant differences were found between normal and excess BMI groups for all body fat composition parameters (*P*<0.05).

**Table 3:** Variations of VAT among slices at different anatomical locations and TVAT.

Variables	All	Women	Men	*P* value*
VAT at the upper pole of kidney (cm^2^)	140.7 ± 79.7	106.9 ± 57.0	165.6 ± 84.9	<0.001*
VAT at the hilum of kidney (cm^2^)	171.2 ± 79.0	138.4 ± 59.3	195.3 ± 83.0	<0.001*
VAT at the lower pole of kidney (cm^2^)	165.6 ± 76.6	140.8 ± 63.1	184.0 ± 80.6	<0.001*
VAT at L2/L3 (cm^2^)	168.9 ± 77.9	137.5 ± 58.3	192.1 ± 82.4	<0.001*
VAT at umbilicus (cm^2^)	146.4 ± 63.0	127.3 ± 48.2	160.5 ± 68.8	<0.001*
VAT at L5/S1 (cm^2^)	120.2 ± 38.9	112.9 ± 34.3	125.6 ± 41.3	0.004*
TVAT (mL)	2176.6 ± 1178.0	1761.5 ± 876.4	2482.6 ± 1276.5	<0.001*

P* for difference between gender groups; VAT, visceral adiposity fat; TVAT, total visceral adiposity fat.

**Table 4:** Differences in VAT area and TVAT volume in different gender and BMI group.

Characteristics	Women			Men		
	Normal BMI	Excess BMI	*P* value*	Normal BMI	Excess BMI	*P* value*
N (%)	94 (30.2)	38 (12.2)	–	107 (34.4)	72 (23.2)	–
BMI (kg/m^2^)	22.0 ± 1.8 (18.8-24.9)	27.2 ± 1.6 (25.1-31.2)	<0.05*	22.0 ± 1.9 (18.6-24.9)	27.5 ± 1.8 (25.1-32.9)	<0.05*
WC (cm)	82.2 ± 7.8	93.1 ± 6.7	<0.05*	84.7 ± 8.2	97.3 ± 7.1	<0.05*
VAT area measured at the upper pole of kidney (cm^2^)	93.5 ± 54.2	140.1 ± 50.5	<0.05*	135.8 ± 69.3	210.0 ± 87.0	<0.05*
VAT area measured at the hilum of kidney (cm^2^)	127.3 ± 60.0	166.0 ± 47.8	<0.05*	165.5 ± 72.7	239.7 ± 77.9	<0.05*
VAT area measured at the lower pole of kidney (cm^2^)	126.2 ± 60.0	176.8 ± 57.2	<0.05*	154.5 ± 63.4	227.7 ± 84.0	<0.05*
VAT area measured at the L2/L3 (cm^2^)	124.6 ± 57.6	169.2 ± 47.3	<0.05*	165.3 ± 68.6	231.8 ± 85.5	<0.05*
VAT area measured at the umbilicus (cm^2^)	117.6 ± 46.8	151.3 ± 43.4	<0.05*	136.7 ± 51.7	195.9 ± 75.9	<0.05*
VAT area measured at the L5/S1 (cm^2^)	107.5 ± 33.3	126.2 ± 33.5	0.05*	114.6 ± 34.7	141.8 ± 44.9	<0.05*
TVAT (mL)	1544.7 ± 816.8	2298.0 ± 792.2	<0.05*	1955.1 ± 1011.8	3266.5 ± 1230.5	<0.05*

*P for difference between gender groups; A BMI of ≤ 24.9kg/m^2^ was considered normal BMI and >25kg/m^2^ was defined as excess BMI.

BMI, body mass index; WC, waist circumference; VAT, visceral adiposity fat; TVAT, total visceral adiposity fat.

### Correlation Between VAT Area of Different Anatomical Landmarks and Total VAT Volume

**Figure 3:**
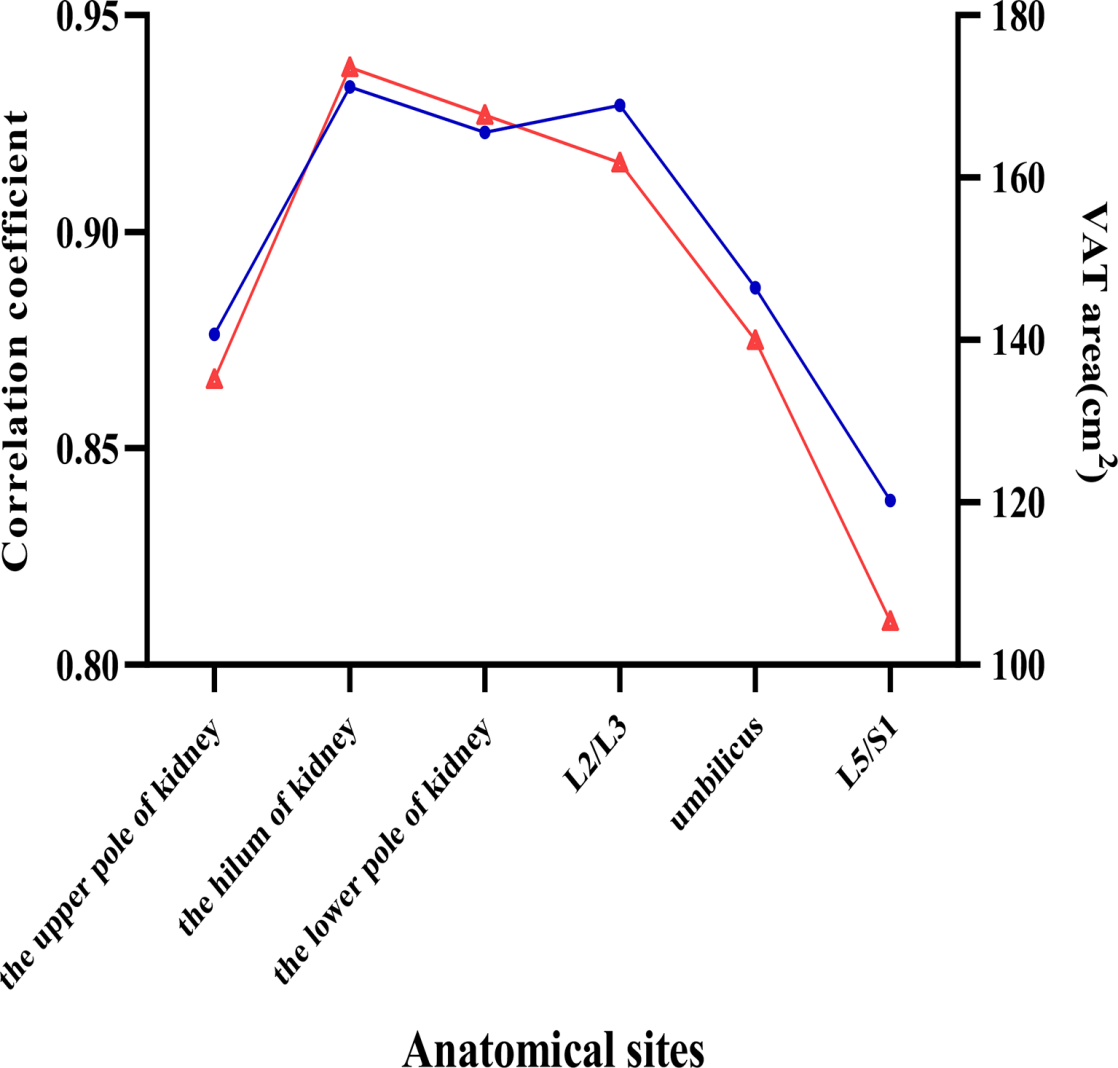
Visceral adipose tissue (VAT) slice areas (red triangle line) and correlation coefficients (blue circle line) of VAT area and total VAT volume at different anatomic locations. Six anatomical sites in the abdomen were selected to investigate the relations between VAT area and total VAT volume.

Linear correlations indicated a strong positively correlation between the single-slice VAT area at different anatomic landmarks and TVAT volumes for both men and women (**Table 5**). Pearson’s correlation coefficients between the VAT area and TVAT volume were both highest at the hilum of kidney level in men (*r*=0.938, *P*<0.001) and women (*r*=0.916, *P*<0.001), but the highest VAT area in women was observed at the lower pole of kidney (*P*<0.001, **Figure 3**).

**Table 5:** The Pearson’s correlation of measurement between a single slice with the total fat volume.

	Correlation coefficients					
	The upper pole of kidney	The hilum of kidney	The lower pole of kidney	L2/L3	Umbilicus	L5/S1
All	0.866	0.938	0.927	0.916	0.875	0.810
Women	0.782	0.916	0.883	0.907	0.840	0.808
Men	0.875	0.938	0.937	0.907	0.874	0.815

All correlation coefficients are significant at P <0.001.

### Estimation of Total VAT Volume From a Single-Slice VAT Area Measurement

**Figure 4:**
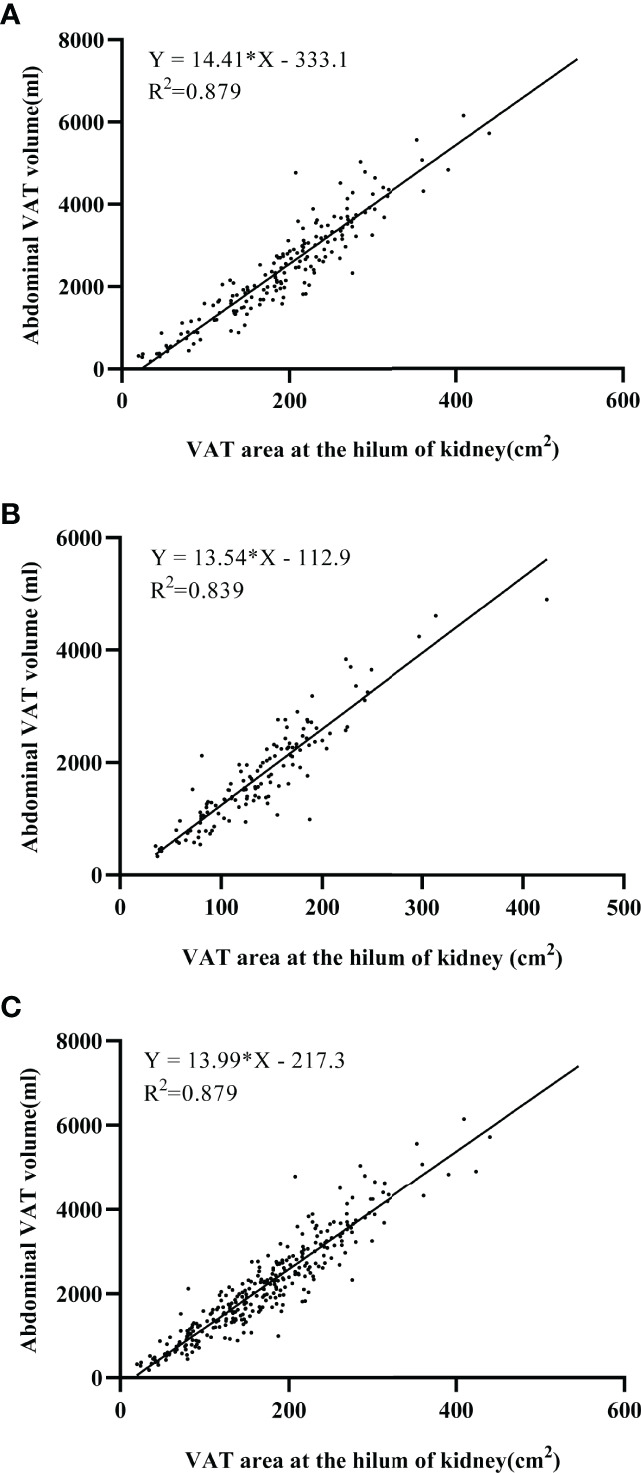
Linear regression models between abdominopelvic visceral adipose tissue (VAT) volumes and VAT area at the hilum of kidney in men **(A)** and in women **(B)** and in all participants **(C)**. Both the coefficient for VAT area and the intercept of the equation are significant (*P* < 0.001). Noted that both scatter plots were well fitted in prediction equations (R^2^ = 0.839-0.879).

Based on linear regression models, we proposed a prediction of TVAT volume using the VAT area at the hilum of kidney level and assessed the effect of covariates on the prediction equations. The scatter plots showed good linearity between the VAT area at the hilum of kidney and abdominopelvic VAT volume in both men (R^2 =^ 0.879, *P*<0.001, **Figure 4**) and women (R^2 =^ 0.839, *P*< 0.001, **Figure 4**). The VAT area measured at the hilum of kidney explains 87.9% of the variance of abdominopelvic TVAT volume with the standard error of estimates (SEE) at 385.4mL. The optimal landmark for measuring VAT area was selected in the multiple regression models as the primary independent variable and abdominopelvic TVAT volume was used as the dependent variable. The contributions of significant covariates (e.g., BMI, WC, and age) to the developed linear model showed improvement of 1.4% in R^2^. The normalized regression equation for total volumes (V) of VAT from the slice area (A) at the hilum of kidney level was as follows.


$$\begin{array}{l} V_{TVAT}\left(mL\right)=0.833\times A\left(cm^{2}\right)+0.076\times BMI\left(kg/m^{2}\right)+0.090\times WC\left(cm\right)-0.042\times age\left(years\right)\\ \left(R^{2}=0.893,\;SEE=385.4mL\right) \end{array}$$

## Discussion

Our study investigated the optimal landmark for single-slice area of visceral adipose tissue to quantify the total VAT volume and the correlations between VAT area and total abdominopelvic VAT volume in QCT scan. From the perspective of research design, many previous studies evaluated the volume of TVAT with VAT area and slice-to-slice interval on abdominal CT or MR images (11, 12, 15, 24, 26). However, there may be imprecise identification of the exact location with the highest correlation for not using continuous scan (11). In this study, using attenuation-based segmentation can accurately quantify the actual overall volume of VAT *via* Frontier cardiac risk assessment technique. Even though most of the previous studies specifically adopted locations with vertebral body and how many centimeters above the vertebral body to perform an examination on the abdomen visceral adipose (11, 15, 26), it’s not practical in clinics using axial images only. Hence, we selected these axial images with more easy-detecting anatomic landmarks that could also represent the fat distribution of abdomen fat. Cheng et al. (15) suggested choosing the L2/L3 as the optimal single slice for visceral fat volume quantification. Thus, we considered to select the L2/L3 as one of the anatomic landmarks. At the same time, the hilum of kidney nears the L2/L3 level, which indirectly reflects the hilum of kidney has a good performance on TVAT volume quantification.

Our results indicated that the VAT area measured at the hilum of kidney have a strong correlation with total abdominopelvic VAT volume in men (*r*=0.938, *P*<0.001) and women (*r*=0.916, *P*<0.001). It may have important clinical implications for BMD quantification patients to apply QCT opportunistic scanning on visceral obesity assessment. Cheng et al. (15) researched the optimal anatomic site for a single slice to estimate the total volume of VAT on single axial QCT images in 389 healthy Chinese subjects, suggesting a great feasibility to measure the VAT area on a single slice at L2/L3 level for estimate the total VAT volume (*r*=0.980, *P*<0.001). Demerath et al. (18) researched the approximation of total VAT with a single MR images in 820 healthy white and black adults and found that the VAT area at the 6 cm above the L4/L5 level can accurately predict total VAT volume. Similarly, Schweitzer and colleges validated the best reference site for a single MRI slice to assess whole-body skeletal muscle and adipose tissue volumes in 142 healthy Caucasian adults and concluded that a single scan at the level of L3 was the optimal site to assess total VAT volume (27). Those results are different from conventional body composition imaging methods that assumed the L4/L5 to have maximum correlation to represent TVAT volume (28). This discrepancy in the optimal site between studies may be explained by the variance between different body sizes and ethnicities (29, 30).

Similar results to Cheng et al. (15), the current study found that the dominate contributor covariate is the VAT area at the hilum of kidney (R^2 =^ 0.879). Covariates of the BMI, WC, and age have a significant effect on the total VAT volume, although these covariates all together make slightly effect with elevated explained variance of merely 1.4%. Thus, VAT measured at the hilum of kidney may potentially represent the total VAT volume, can be applied to the opportunistic screening across a wide range of Chinese healthy subjects. However, the SEE for predicting TVAT volume in individuals from a single-slice area is 385.4mL. This regression equation predict from the VAT area in an individual may cause errors. Therefore, the use of single-slice VAT area to estimate TVAT volume in an individual subject has its limitations.

Our analysis revealed that visceral fat accumulation variance not only in genders but also in different BMI groups (all *P*<0.05). The abdominopelvic visceral fat contribution was remarkably higher in the excess BMI group than normal BMI group. Although the VAT area at each slice varied greatly between men and women, there are no significant differences in BMI and WC between two groups. These findings are consistent with previous studies on central body fat distribution (2, 5, 31).

This study also has some limitations. Firstly, although we found that VAT areas measured at the hilum of kidney have the highest correlations with VAT volume in both men and women, we only choose six representative anatomical landmarks instead of successive landmarks in this study. We prefer to find a more practical anatomical landmark for quantify TVAT volume in clinical use. For that purpose, six identifiable and easy-detecting landmarks are selected in this study. Secondly, we collected these QCT images in two scanners, but the QCT-measured VAT area difference between equipment may be relatively small. Thirdly, this study is a single-center study and including participants only in Henan province, which may not be presentative of the general population in China. The sample contain fewer female participants (132 female) than male (179 male). Further study is needed to validate our findings in multi-center data. Finally, we did not assess the SAT in different groups and the relationship between SAT and total SAT in this study. Although the precise role of visceral fat in metabolic disease have been presented (21, 22), the evaluation of SAT may also be of potential value, we will evaluate SAT in further study. The individual anatomical variation should be considered as a potential element that affecting the selection of the optimal site, it is evitable to some extent.

In conclusion, with QCT opportunistic scanning, the VAT area of the hilum of the kidney in both genders show predictive value in quantifying TVAT volume. Therefore, quantitative CT images in the hilum of kidney could be seen as a reliable site to predict TVAT volume and use this as a marker in clinical practice.

## Data Availability Statement

The original contributions presented in the study are included in the article/supplementary material. Further inquiries can be directed to the corresponding author.

## Ethics Statement

The studies involving human participants were reviewed and approved by Department of Radiology, The First Affiliated Hospital of Zhengzhou University. The patients/participants provided their written informed consent to participate in this study.

## Author Contributions

(I) Conception and design: SC, YJ. (II) Administrative support: JG, YW. (III) Provision of study materials or patients: SC, DM, DS. (IV) Collection and assembly of data: SC, YL, XY, DM, DS. (V) Data analysis and interpretation: YL. (VI) Manuscript writing: All authors. (VII) Final approval of manuscript: All authors.

## Conflict of Interest

The authors declare that the research was conducted in the absence of any commercial or financial relationships that could be construed as a potential conflict of interest.

## Publisher’s Note

All claims expressed in this article are solely those of the authors and do not necessarily represent those of their affiliated organizations, or those of the publisher, the editors and the reviewers. Any product that may be evaluated in this article, or claim that may be made by its manufacturer, is not guaranteed or endorsed by the publisher.
