# Corrigendum: The optimal axial anatomical site for a single-slice area to quantify the total volume of visceral adipose tissue in quantitative CT

**DOI:** 10.3389/fendo.2022.1039351

**Published:** 2022-12-13

**Authors:** Sihui Chen, Duoshan Ma, Danyang Su, Yali Li, Xi Yu, Yaojun Jiang, Jianbo Gao, Yan Wu

**Affiliations:** Department of Radiology, The First Affiliated Hospital of Zhengzhou University, Zhengzhou, China

**Keywords:** optimal anatomical axial site, volume prediction equation, visceral adipose tissue, QCT, total VAT

In the published article, there were some errors in **Table 1** as published. The **Table 1** for the Pitch of Brilliance iCT Elite was displayed as “11” and of Somatom Force was “1”, the Reconstruction kernel of Somatom Force was displayed as “Standard”, and the DFOV was displayed as “250”. The correct statement is that the Pitch of Brilliance iCT Elite is “0.914” and of Somatom Force is “0.8”, the Reconstruction kernel of Somatom Force is “Br40”, and the DFOV for 2 CTs are “350”, etc. The corrected **Table 1** and its caption appear below.

**Table 1:** Summary of acquisition parameters of 2 CT scanners.

CT parameters	Brilliance iCT Elite	Somatom Force
Tube voltage (kV)	120	120
Tube current-time product (mAs)	automatic current selection (DoseRight Index-23)	anatomic tube current modulation (CARE Dose 4D)
Pitch (approximate number)	0.914	0.8
Detector configuration (mm)	128×0.625	192×0.6
Matrix size	512×512	512×512
Slice thickness/increment (mm)	1.0/1.0	1.0/1.0
Reconstruction kernel	Standard	Br40
DFOV (mm)	350	350
Acquisition mode	Helical	Helical
Gantry rotation times (s)	0.5	0.5

CT, computed tomography; DFOV, display field of view.

The authors apologize for this error and state that this does not change the scientific conclusions of the article in any way. The original article has been updated.

